# Measuring What Matters: Redesigning Evaluation With Consumers to Understand Engagement in Hand Therapy Using Virtual Reality

**DOI:** 10.1111/hex.70817

**Published:** 2026-08-11

**Authors:** Margo Coffey, Megan Simons, Jessica Taylor, Gail Kingston

**Affiliations:** ^1^ Occupational Therapy Department, Logan General Hospital Metro South Hospital and Health Service Brisbane Queensland Australia; ^2^ Occupational Therapy Department, Queensland Children's Hospital Children's Health Queensland Hospital and Health Service Brisbane Queensland Australia; ^3^ Children's Health Research Centre The University of Queensland, Faculty of Medicine Herston Queensland Australia; ^4^ Consumer Co‐Researcher and Metro South Health Consumer Partner, C/O Occupational Therapy Department Queen Elizabeth II Jubilee Hospital Coopers Plains Queensland Australia; ^5^ College of Healthcare Sciences James Cook University Townsville Queensland Australia; ^6^ Allied Health Governance Unit Townsville Hospital and Health Service Townsville Queensland Australia

**Keywords:** co‐design, consumer engagement, hand therapy, value‐based care, virtual reality

## Abstract

**Introduction:**

Consumer involvement is essential to ensure health service evaluations reflect what matters to those receiving care. This service improvement project describes how the evaluation of virtual reality (VR) in outpatient hand therapy was redesigned in partnership with consumers to better capture engagement with hand therapy.

**Method:**

The evaluation was undertaken in an outpatient hand therapy service. Initial evaluation relied on standardised quantitative measures, which did not address the original purpose of implementing VR in the treatment setting. Through collaboration with consumer partners and research mentors, the evaluation was redesigned using a value‐based care framework (comfort, capability and calm). Questionnaires using open and closed questions were developed in partnership with consumers and administered to both consumers attending the hand therapy unit and clinicians who worked in the unit.

**Results:**

Seven clinicians and 12 consumers participated. The redesigned evaluation identified that VR enhanced engagement with therapy by creating a low‐pressure and engaging environment. These findings would not have been captured in the original evaluation approach. Participants identified contextual factors (physical space, technology reliability, cultural and language considerations, and the importance of ‘hands‐on’ guidance) that influenced the successful use of VR.

**Conclusions:**

Co‐designing the evaluation of the service improvement project fundamentally changed what was measured and understood. This study demonstrates that involving consumers in how we evaluate projects can provide insights about engagement in therapeutic intervention. Partnering with consumers shaped this project and ensured outcomes reflected real‐world needs. This approach is encouraged across future hand therapy projects and research to ensure that innovation in clinical practice truly aligns with the people they are designed to support.

## 1 Introduction

Virtual reality (VR) has become an accepted modality when used as an adjunct to usual rehabilitation in hand therapy [1, 2, 3]. The use of VR in neurological and vestibular rehabilitation, pain, burns and orthopaedic caseloads has been shown to improve outcomes in range of motion, function and day‐to‐day work‐related tasks [4, 5, 6, 7]. VR promotes engagement with therapy sessions, providing encouragement and positive feedback during rehabilitation, to support patients to adhere to their rehabilitation routines [1].

Evaluation of interventions in hand therapy often focuses on quantitative measures, such as the recovery of physical function [8]. Moreover, despite increasing use of VR in the hand therapy space, evaluation has largely focused on clinical outcomes and technology performance, with limited attention to how patients experience and engage with therapy. A holistic approach to measuring outcomes, however, supports the exploration of how hand therapy interventions can impact on quality of life and psychosocial experience for the consumer and the health professional [9, 10]. Consumer involvement offers an opportunity to address this gap by ensuring that evaluation reflects outcomes that matter to patients, including motivation, confidence and participation in care.

In April 2023, the hand therapy team at Logan General Hospital (LGH), a metropolitan public hospital in Brisbane, Australia, that delivers moderate complexity hand surgery (addressing both elective and urgent clinical needs), commenced a service improvement project using VR as a therapeutic modality to engage patients in their hand rehabilitation. In this setting, hand therapy is an acute (post‐operative) outpatient service for rehabilitation of trauma distal to the mid humerus, for a culturally and linguistically diverse population.

Commercially available games as part of the VR suite were chosen by the clinicians to provide a fun and graded approach to develop and improve impairments in range of motion, coordination and proprioception. Clinicians (OTs and PTs) attended a ‘train the trainer’ session in the use of the VR provided by a local video technology company to ensure the sustainability of trained staff. A step‐by‐step process was documented to set up the environment and the technology prior to each therapy session. VR sessions ran for 15 minutes [11] to minimise any risk to our consumers, within the traditional 30‐minute hand therapy appointment. Commercially available games that did not include teleporting and limited immersion were chosen to prevent cybersickness. Potential incidents of cybersickness or adverse events were recorded in a statewide online register designed to capture data associated with incident relating to clinical care.

The initial evaluation approach was clinician‐led and used tools that had been developed to assess the quality and usability of software and hardware, or measure, quantify and assess the severity of motion or cybersickness symptoms in virtual environments and flight simulators [12, 13, 14]. However, the team realised that the questionnaires failed to address the reason why they had chosen to introduce VR. This prompted a redesign of the evaluation framework, shifting the focus from measuring technology performance or clinical symptoms from the use of VR to understanding patient engagement and experience.

The International Association for Public Participation Australasia [15] outlines levels of consumer engagement at the preparation, execution and translation phases of research. Engagement can include consumers being informed, consulted and involved. Collaborative, leadership and empowerment levels provide consumers with an equal level of influence in the research process, and they can be listed as co‐ or principal investigators [16]. In the current project, consumers were empowered with formal decision‐making authority and helped determine priorities and success criteria (e.g., consumer‐defined outcomes guiding service evaluation).

The aim of this study is to both describe a co‐design process with consumers to reshape the evaluation of a service improvement project and to explore clinicians' and consumers' experience of commercially available VR in a hospital outpatient hand therapy setting.

## 2 Methods

### 2.1 Co‐Design Process

The evaluation of the service improvement project was supported by the Queensland Health Professional Innovators and Leaders in Occupational Therapy (QPILOT) Research Collaborative (herein called the Collaborative). Mentors (comprised of occupational therapists and consumer partners experienced in research) provided perspectives, advice and guidance to occupational therapy clinicians who sought to transition their quality improvement projects to publication or research. This initiative acknowledges that consumers have an important role through co‐design and evaluation of health service improvement projects [17].

Early discussions with the Collaborative identified that the proposed evaluation tools did not capture meaningful aspects of patient experience. Through iterative consultation, regular meetings and feedback loops, consumers prioritised outcomes relating to engagement, including enjoyment, psychological safety and perceived progress. Consumers contributed to critiquing the original evaluation plan, identifying outcome domains, shaping survey questions and language and interpreting findings. This process resulted in the adoption of the 3C's framework (confirm, capability and calm) [18], which aligned with consumer priorities and provided a structure for evaluating engagement.

A convenience sample of all consumers from the existing hand therapy outpatients who had used VR during a hand therapy session were invited to participate for the purpose of conducting a service evaluation. An information sheet was developed by our consumer partner to encourage open communication and active consumer involvement. There were no formal exclusion criteria beyond the ability to participate in VR as part of their usual treatment session and willingness to complete a survey. Clinician participants were also invited to review their experience and knowledge of VR and included both Occupational Therapists and Physiotherapists working in the study setting.

An ethics exemption was granted EX/2024/QMS/112961 (31 Oct Ver 1). In accordance with the National Health and Medical Research Council National Statement on Ethical Conduct in Human Research, the local Human Research Ethics Committee (HREC) review determined the project to be exempt from full ethical review. This project involved analysis of de‐identified survey data, posed negligible risk and was conducted for service evaluation and improvement purposes. The use of VR is acknowledged as an accepted clinical intervention, and the collection of survey data provided an evaluation of the impact of VR in a real‐world setting, posing minimal burden to participants [19]. Consumers were free to decline VR as part of their therapy session and not to complete the survey. The Standards for Quality Improvement Reporting Excellence (SQUIRE) 2.0 were used for manuscript preparation [20].

### 2.2 Evaluation Questionnaires

A value‐based care approach, which explored consumers' perspectives, collected information to reflect what mattered most to them and asked, ‘how are you doing?’ as opposed to ‘how are we doing?’ [21]. Two questionnaires were developed specifically for the evaluation by the research team for the purpose of addressing the aim. Questions were a mix of closed, 5‐point Likert scales, dichotomous yes/no and open‐ended questions. The questionnaire was piloted with consumers and clinical staff and to test the suitability and readability. Minor feedback was provided.

Consumer participants explored the concepts of calm (measures the extent to which consumers can continue their life during care), capability (measures a person's functional status) and comfort (relief from physical and emotional pain) [17]. Clinician participants recorded aspects related to the implementation of VR [22], and both answered questions addressing dimensions of clinical utility [23] for integration of VR into hand therapy treatment sessions (Table 1).

**Table 1:** Questions answered by clinicians and consumers.

Questions for clinicians (pre)	Questions for clinicians (post)	Questions for consumers
How long have you worked in hand therapy?	Do you think VR was beneficial to your patient population?	How satisfied are you that VR therapy assists you to move your hand more comfortably?
Have you had any experience of VR prior to this project? If yes where?	Please rate the quality of the VR experience at LGH?	How do you think VR could support you in returning to your daily activities?
Do you think VR would be beneficial to your patient population?	What factors influenced your above response?	How would you rate your comfort during the session?
Have you reviewed any literature regarding the use of VR as a therapeutic modality?	Do you feel confident using VR as part of your treatment in the LGH clinical setting?	How could we improve the comfort for you?
Do you feel confident using VR as part of your treatment in the LGH clinical setting?	How easy do you think it would be to implement VR into your routine practice?	Did you enjoy the session?
How easy do you think it would be to implement VR into your routine?	Do you have any recommendations for improving VR use in this setting?	Do you have any other comments or feedback you would like to share?

### 2.3 Data Analysis

Descriptive statistics were used to summarise survey responses, which was consistent with the study aim of providing insight into consumer and clinical experiences. A qualitative content analysis of open‐ended survey questions was undertaken using a deductive approach guided by Smart's clinical utility framework [24]. Trustworthiness was enhanced through strategies addressing credibility, dependability, confirmability and transferability. These included peer review of coding and interpretation, use of a structured analytical framework and involvement of multiple researchers and partners in data interpretation, which helped to ensure findings were grounded in participant perspectives [25]. Specifically, coding categories were based on the three concepts of value‐based care (care, comfort and calm), and open‐ended questionnaire data were coded into these predetermined categories. The initial coding was conducted individually by all four research team members who then met to discuss and come to agreement on the organisation and presentation of the data.

## 3 Results

The redesigned evaluation informed by consumer partnership enabled identification of engagement‐related outcomes that were not captured in the initial evaluation approach. A total of 7 clinicians (5 occupational therapists and 2 physiotherapists) and 12 consumers participated in the VR session and survey evaluation. The clinician participants had an average of 8.5 years of clinical experience in hand therapy and self‐reported very limited experience with VR at the commencement of the study. The consumer participants consisted of adults aged 18–75 years who were residing in the local district and receiving hand therapy services at the time of data collection. Consumers had been referred to the hand therapy service following upper limb injuries, including phalangeal fractures of the fingers and thumb, metacarpal fractures, wrist fractures and tendon repairs. No interpreters or alternative communication strategies were used in this study.

All consumer participants (100%, *n* = 12) reported they enjoyed the VR session, despite ‘the VR seemed to have a few bugs’ (Consumer 2). As one consumer stated ‘It was amazing to see technology being used to aid in recovery. The automatic feedback and correction resonates, and I am glad I could participate’ (Consumer 5). However, cultural, linguistic or digital literacy barriers must always be considered. Another stated ‘If you had VR at home, it would be good practice. I prefer the face‐to‐face exercises with the therapist’ (Consumer 2) and a suggestion for ‘hands on correction‐ move my arm and shoulder to demonstrate the correction’ (Consumer 5).

### 3.1 Capability

All consumers reported improvement in their hand flexibility, range, mobility, coordination and movement:

In 15 minutes, I was able to make a fist which I could not do before.

(Consumer 9)

I was competitive and not thinking about my finger, while the mobility in my finger was improving.

(Consumer 4)

### 3.2 Comfort

Most (83.3% *n* = 10) of the consumers reported the VR session was very comfortable with the remaining 16.7% (*n* = 2) reporting it was moderately comfortable. Consumers identified additional strategies to improve their comfort:

Very comfortable. Perhaps it would be more convenient for the doctor (physiotherapists) and patients to work in a larger room.

(Consumer 3)

More time to adjust.

(Consumer 9)

It took me a minute to understand the physics.

(Consumer 4)

Need to have another level but it was good. I don't pick up tech easily but I got the hang of it pretty quick.

(Consumer 8)

### 3.3 Calm

Benefits associated with the calm component included:

(VR) allows me to use my hand without the pressure to do tasks.

(Consumer 8)

It put me in a low stress environment providing real life feedback.

(Consumer 5)

One participant reported VR helped to ‘… remove the psychological block that is preventing me from making better progress’ (Consumer 3).

### 3.4 Clinical Utility

#### 3.4.1 Appropriate

Prior to the participation in this project, all (100%, *n* = 7) of the clinician participants believed VR would be appropriate for their consumers. This remained unchanged throughout the project. 70% (*n* = 5) of the clinician participants had reviewed literature regarding the use of VR as a therapeutic modality prior to the commencement of this project. No adverse events such as cybersickness were recorded during data collection.

#### 3.4.2 Accessible and Practicable

The technology (hardware and software) was purchased for AUD$10,230. This included 2x Oculus Quest headsets, a premium ipad, TV screen, wall mount and the costs associated with linking with the local tech provider (for training and support). The train‐the‐trainer approach helped to ensure sustainability of the use of VR amongst clinicians. Clinicians preferred to utilise the therapy assistant to set up and run the VR sessions, thus ensuring all components were adequately charged prior to commencement of a treatment session.

#### 3.4.3 Acceptability

Clinician participants (100%, *n* = 7) rated the quality of the VR experience as ‘very high’ to ‘moderately high’. Improvements recommended were increased space, high‐speed internet connection and access to a corporate credit card to purchase more VR games. One consumer had suggested ‘a golf game’ (Consumer 11), which aligned to their leisure interest. Confidence of the clinician (at baseline) to use VR in standard care, a lack of speed of internet utilised to cast the visual images to the TV screen, minimal experience with the VR technology (both consumer and clinician) and positioning of the goggles for some consumers raised challenges. Despite this, the overwhelming positive feedback and enablers included enthusiasm from participants for the VR modality and fun for both consumers and clinicians.

## 4 Discussion

This study demonstrated how consumer and public involvement can fundamentally reshape evaluation in health service improvement. Rather than simply contributing to data collection, our consumers in this project influenced what was measured, how it was measured and ultimately whether the intervention addressed the aim we had originally intended. Prior to consumer engagement for co‐design, the proposed evaluation relied solely on standardised quantitative questionnaires. While these tools are widely used [26, 27, 28, 29], they do not always capture the emotional, motivational or practical dimensions of rehabilitation that matter most to people.

The shift to a value‐based care approach, guided by the 3Cs (calm, capability and comfort), enabled the team to explore how consumers experienced VR—how safe they felt, whether the session reduced stress, whether it built confidence, and how it influenced their sense of progress and recovery. For many participants, VR created an environment where therapy felt less pressured and more engaging, which is an outcome that may not be visible in biomechanical data but is critical for adherence and long‐term improvement.

Consumers also identified contextual factors that influence the successful use of VR, such as physical space, technology reliability, cultural and language considerations and the importance of ‘hands‐on’ guidance from the clinicians at this facility. These insights emphasise that VR is not just a tool but part of a broader therapeutic experience.

These findings support earlier calls for greater appreciation of a holistic approach to assessment, intervention and evaluation. Valdes states that few hand therapist evaluations address outcomes related to psychosocial issues and recommends the use of more than one measure in areas that are important to the consumer and outside the traditional disease or region‐specific measures [30]. Qualitative information provides important information in patient‐reported experience measures to allow relevant and contextual changes [31].

A recent international survey of hand therapists (*n* = 236 respondents from 30 countries) identified low uptake of VR as a therapeutic modality, with high costs and low understanding as two major barriers. The findings from the current study can inform how consumer feedback will shape future implementation support. Consumers reported benefit from using commercially available games, which may reduce the burden of high entry and maintenance costs. ‘Measuring what matters’ has enhanced understanding of the consumer challenges and engagement using VR beyond commonly used patient‐reported experience measures including pain, anxiety and satisfaction [32]. Study findings support high clinical utility for a hospital catchment characterised by a younger, more culturally diverse population with comparatively lower income, lower educational attainment and higher unemployment than (removed for peer review) averages, alongside a higher proportion of rental housing [33].

In the rapidly expanding and developing world of technology, VR is a tool that can be harnessed and adapted to suit the relevant client group. It has an important place in the future of healthcare, as it provides a fun and engaging way to complete rehabilitation. As one of our consumers suggested, it may be a useful tool for further use in home exercise programs as affordability and accessibility to VR improve. This project used commercially available VR games as a considerable cost saving when compared to the games developed for a specific clinical population such as ‘A Wanderer's Tale’ [34] and ‘Snow World’ [35]. In the future, the use of Artificial Intelligence may enable a customised VR game to emulate the individual's preferred occupation/s. This would support the consumer (with a biceps repair) who expressed his preference for a golf‐based VR game.

Hand therapy research until now has predominantly involved consumers at the consult or involvement level [14], giving feedback on the research topic and data gathering tools, checking data and data analysis [36, 37, 38]. Consumers in these studies were involved in the development phase to consult and advise for items for inclusion in patient‐reported outcome measures (PROMs). Interviews were conducted to understand what outcomes mattered to consumers; however, no consumers were listed as members of the research team. Partnering with consumers shaped the current project significantly and ensured that the outcomes reflected real‐world needs. This approach should be encouraged across future hand therapy and occupational therapy research to ensure that innovations truly align with the people they are designed to support.

This study has several limitations that should be considered when interpreting the findings. This was a small, single‐site service evaluation with a convenience sample, which limits the generalisability of the findings beyond the local clinical context to the broader population. The possibility of selection bias was addressed by inviting all current consumers of the hand therapy outpatient service, whilst response bias was minimised by assuring surveys were anonymous, though the research team acknowledge this was limited due to the small number of respondents. The lead researcher was based in the facility and involved in recruitment of the participant, another potential source of bias. This was managed by the involvement of all research team members undertaking coding of open‐ended response data and checking survey responses. The evaluation utilised co‐designed questionnaires that were not psychometrically validated. While this approach was intentional to capture consumer‐defined outcomes, it limits the reliability, reproducibility and comparability of findings with existing literature. This study was not designed to assess effectiveness, and no control group or pre‐post comparison was included. As such, no causal inferences can be made regarding the impact of VR on clinical or experiential outcomes. Important clinical variables such as injury severity, rehabilitation stage, pain levels and baseline function were not controlled for, reflecting the real‐world nature of the service evaluation but introducing potential confounding. Finally, qualitative responses were brief, which may reflect time constraints, survey format or the culturally and linguistically diverse population. Future research incorporating interviews or mixed qualitative approaches may provide richer insight.

Despite these limitations, the study provides important insights into how consumer partnership can reshape evaluation design and priorities in clinical practice.

## 5 Conclusion

Co‐designing the evaluation of the service improvement project fundamentally changed what was measured and understood. This study demonstrates that involving consumers in how we evaluate projects can provide insights about engagement in therapeutic intervention. Partnering with consumers shaped this project and ensured outcomes reflected real‐world needs. This approach is encouraged across future hand therapy projects and research to ensure that innovation in clinical practice truly aligns with the people they are designed to support.

## 6 Consumer or Public Contribution

Consumers were engaged at the empowerment level, meaning they were active partners in shaping the study. They contributed to identifying the limitations of the initial evaluation approach, co‐designed the revised evaluation framework and helped define outcomes that reflected what mattered most to people receiving hand therapy. Consumer partners were actively involved in interpreting finding and shaping the narrative of this manuscript. They helped to shift the focus of the study from technology evaluation to understanding engagement in therapy, demonstrating the impact of meaningful partnership on research design and outcomes.

## Author Contributions

**Margo Coffey:** conceptualization, investigation, funding acquisition, writing – original draft, methodology, writing – review and editing, software, formal analysis, project administration. **Megan Simons:** conceptualization, investigation, writing – original draft, methodology, writing – review and editing, formal analysis, supervision. **Jessica Taylor:** conceptualization, investigation, writing – original draft, methodology, writing – review and editing, resources. **Gail Kingston:** conceptualization, investigation, writing – original draft, methodology, writing – review and editing, formal analysis, supervision.

## Ethics Statement

An ethics exemption was granted EX/2024/QMS/112961 (31 Oct Ver 1). In accordance with the National Health and Medical Research Council (NHMRC) National Statement on Ethical Conduct in Human Research, the local Human Research Ethics Committee (HREC) review determined the project to be exempt from full ethical review.

## Conflicts of Interest

The authors declare no conflicts of interest.

## Data Availability

The data that support the findings of this study are available on request from the corresponding author. The data are not publicly available due to privacy or ethical restrictions.
